# Frailty Index Laboratory (FI-Lab) as predictor of poor outcomes for intra-hospital multidrug-resistant *Klebsiella pneumoniae* bloodstream infections: a single-centre retrospective cohort study

**DOI:** 10.1093/jac/dkaf359

**Published:** 2025-10-07

**Authors:** Carmen Pellegrino, Nicola Veronese, Laura De Santis, Angela Amendolara, Giorgia Manco Cesari, Vincenzo Giliberti, Mariangela Cormio, Alessia Lugli, Marinella Cibelli, Giuliana Metrangolo, Giulia Patti, Annunziata Ilenia Ritacco, Valentina Totaro, Luigi Ronga, Giacomo Guido, Luisa Frallonardo, Elda De Vita, Maria Chironna, Francesco Di Gennaro, Annalisa Saracino

**Affiliations:** Clinic of Infectious Diseases, Department of Precision and Regenerative Medicine and Ionian Area (DiMePRe-J), University of Bari Aldo Moro, Bari 70124, Italy; Faculty of Medicine, Saint Camillus International University of Health Sciences, Rome, Italy; Clinic of Infectious Diseases, Department of Precision and Regenerative Medicine and Ionian Area (DiMePRe-J), University of Bari Aldo Moro, Bari 70124, Italy; Clinic of Infectious Diseases, Department of Precision and Regenerative Medicine and Ionian Area (DiMePRe-J), University of Bari Aldo Moro, Bari 70124, Italy; Clinic of Infectious Diseases, Department of Precision and Regenerative Medicine and Ionian Area (DiMePRe-J), University of Bari Aldo Moro, Bari 70124, Italy; Clinic of Infectious Diseases, Department of Precision and Regenerative Medicine and Ionian Area (DiMePRe-J), University of Bari Aldo Moro, Bari 70124, Italy; Clinic of Infectious Diseases, Department of Precision and Regenerative Medicine and Ionian Area (DiMePRe-J), University of Bari Aldo Moro, Bari 70124, Italy; Clinic of Infectious Diseases, Department of Precision and Regenerative Medicine and Ionian Area (DiMePRe-J), University of Bari Aldo Moro, Bari 70124, Italy; Clinic of Infectious Diseases, Department of Precision and Regenerative Medicine and Ionian Area (DiMePRe-J), University of Bari Aldo Moro, Bari 70124, Italy; Clinic of Infectious Diseases, Department of Precision and Regenerative Medicine and Ionian Area (DiMePRe-J), University of Bari Aldo Moro, Bari 70124, Italy; Clinic of Infectious Diseases, Department of Precision and Regenerative Medicine and Ionian Area (DiMePRe-J), University of Bari Aldo Moro, Bari 70124, Italy; Clinic of Infectious Diseases, Department of Precision and Regenerative Medicine and Ionian Area (DiMePRe-J), University of Bari Aldo Moro, Bari 70124, Italy; Clinic of Infectious Diseases, Department of Precision and Regenerative Medicine and Ionian Area (DiMePRe-J), University of Bari Aldo Moro, Bari 70124, Italy; Department of Interdisciplinary Medicine, Hygiene Section, University of Bari, Bari, Italy; Clinic of Infectious Diseases, Department of Precision and Regenerative Medicine and Ionian Area (DiMePRe-J), University of Bari Aldo Moro, Bari 70124, Italy; Clinic of Infectious Diseases, Department of Precision and Regenerative Medicine and Ionian Area (DiMePRe-J), University of Bari Aldo Moro, Bari 70124, Italy; Clinic of Infectious Diseases, Department of Precision and Regenerative Medicine and Ionian Area (DiMePRe-J), University of Bari Aldo Moro, Bari 70124, Italy; Department of Interdisciplinary Medicine, Hygiene Section, University of Bari, Bari, Italy; Clinic of Infectious Diseases, Department of Precision and Regenerative Medicine and Ionian Area (DiMePRe-J), University of Bari Aldo Moro, Bari 70124, Italy; Clinic of Infectious Diseases, Department of Precision and Regenerative Medicine and Ionian Area (DiMePRe-J), University of Bari Aldo Moro, Bari 70124, Italy

## Abstract

**Background and objectives:**

Bloodstream infections (BSIs) caused by carbapenemase-resistant *Klebsiella pneumoniae* (CR-Kp) are associated with high morbidity and mortality, particularly in frail patients. Identifying frailty upon hospital admission may improve clinical decision-making and outcomes. This study evaluates the Frailty Index Laboratory (FI-Lab) as a predictive tool for adverse outcomes in patients with CR-Kp BSIs.

**Methods:**

This retrospective cohort study was conducted between January 2020 and September 2024 at the Policlinico Universitario di Bari. Frailty was assessed using FI-Lab, derived from 35 laboratory parameters measured within 96 h of admission. Outcomes included all-cause mortality, 28 day mortality and relapse, with FI-Lab predictive accuracy evaluated via ROC curve analysis.

**Results:**

A total of 182 patients with CR-Kp BSIs were included, of whom 48 died (26.3%). Non-survivors had significantly higher FI-Lab scores than survivors (0.66 ± 0.10 versus 0.33 ± 0.16, *P* < 0.0001). FI-Lab demonstrated excellent predictive accuracy for mortality (AUC = 0.94), 28 day mortality (AUC = 0.87) and relapse (AUC = 0.87). Each 0.10-point increase in FI-Lab was associated with a higher risk of mortality (HR = 2.07), 28 day mortality (HR = 1.86) and relapse (HR = 1.52).

**Conclusions:**

The FI-Lab proved to be a simple and robust tool for early frailty assessment in patients with CR-Kp BSIs. Further studies are needed to validate these findings and explore the broader applicability of FI-Lab in managing infections in vulnerable populations.

## Introduction

Bloodstream infections (BSIs) caused by multidrug-resistant *Klebsiella pneumoniae* (MDR-Kp) are associated with high morbidity and mortality rates, posing a significant challenge for clinicians worldwide.^1,2^

Treatment options are limited and primarily involve the use of novel beta-lactam beta-lactamase inhibitor (BLBLI) combinations, such as ceftazidime/avibactam, meropenem/vaborbactam or imipenem/relebactam, either as monotherapy or in combination with older agents like aminoglycosides, fosfomycin, colistin or aztreonam.^3–7^ Alternative second-line options, such as cefiderocol,^8^ are typically reserved for patients with limited or no other treatment options, particularly those infected with highly resistant pathogens, including non-fermenting Gram-negative bacteria or NDM-producing strains.^9^

The choice of treatment depends on several factors, including clinical severity, site of infection and underlying resistance mechanisms (e.g. KPC, OXA-48 or MBL).^10–13^

Frailty is an age-related condition characterized by increased vulnerability and diminished physiological reserve, leading to a reduced ability to respond to stressors.^14,15^ It arises from biological and psychological decline, often accompanied by chronic low-grade inflammation and immune hyperactivation, resulting in progressive cellular damage.^14–16^

These processes, commonly referred to as immunosenescence and inflammaging, significantly impact patients’ health and resilience.^16,17^ Frailty has profound implications for both individuals and healthcare systems, making it a critical public health concern.^18^

Among the tools developed to assess frailty, the Frailty Index (FI) based on laboratory test data (FI-Lab) has emerged as a practical and objective method. It leverages routinely collected laboratory parameters to quantify frailty, offering a standardized approach that is less reliant on subjective criteria or time-consuming questionnaires.^19^Studies have demonstrated that the FI-Lab is a significant predictor of adverse health outcomes, including mortality, hospitalization and disability.^20^ This makes it particularly suitable for emergency settings, where rapid, reproducible assessments are essential.^21^ However, data about the use of a FI-Lab in older people affected by BSIs caused by MDR-Kp are still not available, even if these infections may represent an important problem in older patients for which some challenges in treatment (limited antibiotic options, costs of antibiotic treatments, and drug interactions) are commonly present.^22^ KPC-type carbapenemase is the most widespread, with a significant increase in occurrence among elderly due to frequent hospitalizations, heavy antibiotic usage and diminished resistance to infections.^21,23^

Consequently, understanding the condition of frailty may help in therapeutic decision-making.^22^ Given this background, the present study aims to validate the use of the FI-Lab as a predictive tool for therapeutic response and mortality in patients with BSIs caused by MDR-Kp. By providing clinicians with an accessible and easy-to-use method for assessing frailty, this study seeks to enhance clinical decision-making and guide tailored therapeutic strategies, ultimately improving patient outcomes.

## Material and methods

### Participants

Between 1 January 2020 and 30 September 2024, all patients aged 18 years or older hospitalized at the Policlinico Universitario di Bari with a diagnosis of bacteraemia by KPC- and NDM-producing *K. pneumoniae* were enrolled in this study. No additional inclusion criteria were applied to ensure a real-life clinical scenario and capture the full spectrum of cases.

Patient data were retrospectively collected from medical records and included demographic characteristics, clinical history, microbiological findings and treatment details. Carbapenem-resistant Gram-negative bacteraemia was confirmed using antimicrobial susceptibility testing based on the EUCAST guidelines. The study protocol received approval from the Local Ethics Committee on February 2021 (protocol number 02/2021). Patient data were anonymized and managed in accordance with the Declaration of Helsinki and local data protection regulations.

### Exposure: construction of the Frailty Index Laboratory (FI-Lab)

The FI-Lab was developed using 35 laboratory parameters collected within the first 4 days of hospitalization. These parameters included blood count, liver function, renal function, pancreatic function, blood glucose, lipid profile, serum electrolytes, coagulation markers, inflammatory markers and hormonal profiles (including thyroid function and serum vitamin D levels) (Table 1).^21^ Each parameter was categorized as normal (assigned a value of 0) or abnormal (assigned a value of 1). The total number of abnormalities was summed and divided by the number of tests available for each patient, yielding a final score ranging from 0 to 1, with higher scores indicating greater frailty. Patients with fewer than 10 available laboratory tests were excluded and no imputation methods were applied for missing data.

**Table 1.** Laboratory variables for frailty index

	Item	No frailty (normal)*	+1 Frailty risk (abnormal)
Routine blood test			
1	Hb (g/L)	115–150 (F)	<110 or >150 (F)
		130–175 (M)	<130 or >175 (M)
2	PLT (*10^9/L)	101–320	<101 or >320
3	WBC (*10^9/L)	3.5–9.5	<3.5 or >9.5
4	NEUT (*10^9/L)	1.8–6.3	<1.8 or >6.3
5	LYMPH (*10^9/L)	1.1–3.2	<1.1 or >3.2
Hepatic function			
6	TBil (umol/L)	5.0–26.0	<5.0 or >26.0
7	DBil (umol/L)	≤8	>8
8	IDBil (umol/L)	≤20	>20
9	ALT (IU/L)	≤40 (F)	>40 (F)
		≤50(M)	>50(M)
10	AST (IU/L)	≤35 (F)	>35 (F)
		≤40(M)	>40(M)
11	ALB (g/L)	32–55	<32 or >55
12	ALP (IU/L)	35–135 (F)	<35 or >135 (F)
		45–125 (M)	<45 or >125 (M)
13	GGT (IU/L)	7–45 (F)	<7 or >45 (F)
		10–60 (M)	<10 or >60 (M)
14	CK (IU/L)	40–200 (F)	<40 or >200 (F)
		50–310 (M)	<50 or >310 (M)
15	LDH (IU/L)	120–250	<120 or >250
Fast blood glucose			
16	GLU (mmol/L)	3.9–6.11	<3.9 or >6.11
Renal function			
17	CREA (umol/L)	41–81 (F)	<41 or >81 (F)
		57–111(M)	<57 or >111 (M)
Blood lipid			
18	TG (mmol/L)	≤2.3	>2.3
19	CHOL (mmol/L)	≤5.6	>5.6
20	HDL-C (mmol/L)	≥1.15(F)	<1.15(F)
		≥0.9(M)	<0.9(M)
21	LDL-C (mmol/L)	≤4.11	>4.11
Blood electrolyte			
22	NA (mmol/L)	137.0–147.0	<137.0 or >147.0
23	K (mmol/L)	3.5–5.3	<3.5 or >5.3
24	MG (mmol/L)	0.75–1.02	<0.75 or >1.02
25	CA (mmol/L)	2.2–2.7	<2.2 or >2.7
Blood coagulation			
26	INR	0.80–1.30	<0.80 or >1.30
27	Fib (g/L)	2.0–4.0	<2.0 or >4.0
28	ᴅ-dimer	<150	>150
Blood respiratory			
29	pO2 (mmHg)	>60	<60
30	Ph	7.35–7.45	<7.35 or >7.45
31	PCO2 (mmHg)	35–45	<35 or >45
32	Lactates (mmol/L)	0.5–2.2	<0.5 or >2.2
33	P/F ratio	>150	<150
Inflammation			
34	CRP (mg/L)	<5	>5
35	Procalcitonin (pg/mL)	<0.05	>0.05
36	Ferritin (ug/L)	24–300	<24 or >330
Hormones			
37	Vitamin D (nmol/L)	50–150	<50 or >150
38	TSH (mIU/L)	0.4–4.0	<0.4 or >4.0
Pancreas			
39	Amylases (UI/L)	<150	>150
40	Lipases (UI/L)	<150	>150

Abbreviations: F, female; M, male; RBC, red blood cell; Hb, haemoglobin; HCT, haematocrit; MCV, mean corpuscular volume; MCH, mean corpuscular haemoglobin; MCHC, mean corpuscular haemoglobin concentration; RDW-CV, red cell distribution width-coefficient of variation; RDW-SD, red cell distribution width-standard deviation; PLT, platelets; WBC, white blood cell; NEUT, neutrophil; LYMPH, lymphocyte; MONO, monocyte; EO, eosinophil; BASO, basophil; TBil, total bilirubin; DBil, direct bilirubin; IDBil, indirect bilirubin; ALT, alanine transaminase; AST, aspartate aminotransferase; TP, total protein; ALB, albumin; ALP, alkaline phosphatase; GGT, gamma-glutamyl transpeptidase; CK, creatine kinase; LDH, lactate dehydrogenase; GLU, glucose; UREA, urea; CREA, creatinine; URIC, uric acid; TG, triglyceride; CHOL, cholesterol; HDL-C, high-density lipoprotein cholesterol-C; LDL-C, low-density lipoprotein cholesterol-C; NA, sodium; K, potassium; CL, chlorinum; MG, magnesium; CA, calcium; P, phosphorus; PT, prothrombin time; INR, international normalized ratio; APTT, activated partial thromboplastin time; Fib, fibrinogen.

### Outcomes: mortality, 28 day mortality and relapse

The primary outcome was overall mortality, documented through medical records or death certificates. Secondary outcomes included the following:

1. 28 day mortality, defined as death occurring within 28 days of the BSI diagnosis.
2. Relapse, defined as the recurrence of the index organism in blood cultures accompanied by clinical or laboratory deterioration within 28 days after prior eradication.

### Confounders

Potential confounders in the association between FI-Lab and outcomes were assessed. Data collected at hospital admission included the following:

- Demographics: Age and sex
- Clinical parameters:
  - Presence of fever
  - SOFA score: A tool used to evaluate organ dysfunction severity across six systems (respiratory, cardiovascular, hepatic, coagulation, renal and central nervous systems), with scores ranging from 0 to 24. Higher scores indicate more severe dysfunction
  - Presence of septic shock, defined as profound circulatory, cellular and metabolic abnormalities requiring vasopressors to maintain a mean arterial pressure ≥65 mmHg and serum lactate levels >2 mmol/L (>18 mg/dL) in the absence of hypovolaemia^24,25^
- Provenance: Patient origin (home, nursing home or rehabilitation facility)
- Previous medical history: Hospitalization or infections in the three months prior to admission

Antibiotic resistance and therapy data were also collected as follows:

- Resistance genes: Rapid PCR testing (Xpert Carba-5)^26^ identified resistance genes in carbapenem-resistant *K. pneumoniae*. The most common genes detected were KPC and NDM, which were used to stratify patients, as these genes differ in their susceptibility profiles, associated morbidity and mortality rates.^27^
- Antibiotic therapy: Categorized into first-line treatments (ceftazidime/avibactam versus meropenem/vaborbactam) and second-line options (gentamicin/amikacin, fosfomycin or aztreonam).

Comorbidities were summarized using the Charlson Comorbidity Index (CCI),^28^ which was included in the analysis as a confounding factor.

### Statistical analysis

Patients were stratified by survival status (alive versus deceased). Continuous variables were reported as means with standard deviations (SD), while categorical variables were expressed as absolute numbers and percentages. Normality was assessed using the Kolmogorov–Smirnov test. Comparisons were conducted as follows:

- Continuous variables: Student’s *t*-test for independent samples.
- Categorical variables: Chi-squared test, with Fisher’s correction where appropriate.

The association between FI-Lab and mortality was evaluated using Cox regression analysis, adjusted for confounders. Factors with a *P* value <0.05 in univariate analyses were included in the multivariate model. We assessed the collinearity using the variance inflation factor over two as cut-off: however, no one of the factors included was excluded for this reason. Results were reported as hazard ratios (HR) with 95% confidence intervals (95% CI). FI-Lab was analysed incrementally (0.01-point increases), and the cohort was initially divided into two groups (FI-Lab <0.46 versus  ≥ 0.46). However, no patients with a FI-Lab <0.54 died, precluding further subgroup analysis.

The predictive accuracy of FI-Lab for overall mortality, 28 day mortality and relapse was assessed using receiver operating characteristic (ROC) curves, reported as the AUC with 95% CI. The Youden’s index identified the optimal cut-off point for sensitivity and specificity, which was 0.46 for all three outcomes in this cohort.

The interaction between FI-Lab and antibiotic therapy was also analysed. Data were stratified by frailty grade, focusing on frail patients (FI-Lab ≥0.46) due to convergence issues in non-frail groups.

The association between first-line therapies (ceftazidime/avibactam versus meropenem/vaborbactam) and outcomes was analysed, using the latter as the reference group.

All statistical analyses were performed using SPSS 26.0 for Windows (SPSS Inc., Chicago, IL). Statistical tests were two-tailed, and a *P* value <0.05 was considered significant.

## Results

The study analysed 182 patients, divided into survivors (*n* = 134) and dead (*n* = 48). Non-survivors had a significantly higher mean age than survivors (70.3 ± 13.6 years versus 62.8 ± 15.0 years, *P* = 0.002) and presented with a higher SOFA score at the onset of infection (6.9 ± 4.7 versus 3.8 ± 2.5, *P* < 0.0001) (Table 2). The prevalence of shock was also significantly higher among non-survivors (51.1% versus 26.1%, *P* = 0.002), as was the incidence of acute respiratory failure (53.2% versus 30.6%, *P* = 0.004). Regarding comorbidities, dementia was more frequent in non-survivors (31.3% versus 12.8%, *P* = 0.007), as was diabetes with complications (16.7% versus 6.0%, *P* = 0.04) (Table 2). The FI-Lab was markedly higher among non-survivors (0.66 ± 0.10) compared with survivors (0.33 ± 0.16, *P* < 0.0001).

**Table 2.** Baseline characteristics by survival status

Parameter	Alive (*n* = 134)	Dead (*n* = 48)	*P* value
Mean age	62.8 (15.0)	70.3 (13.6)	**0**.**002**
Male gender	65.7	62.5	0.69
*Admission physical examination findings*			
Fever	72.0	71.4	1.00
SOFA score	3.8 (2.5)	6.9 (4.7)	**<0**.**0001**
Shock	26.1	51.1	**0**.**002**
Home	84.3	75.0	0.39
Hospitalization in the previous 3 months	43.3	39.6	0.73
Infection in the previous 3 months	20.9	25.0	0.55
KPC	74.6	72.9	0.82
MBL	25.4	27.1	0.82
Multiple microorganisms	29.1	45.8	**0**.**04**
Antibiotic therapy			0.71
Cefiderocol	3.7	2.1	
Ceftazidime/avibactam	59.7	54.2	
Imipenem/relebactam	0.7	2.1	
Meropenem/vaborbactam	35.8	41.7	
+fosfomycin	23.9	18.8	0.47
+aztreonam	26.1	29.2	0.68
+no other antibiotic	43.3	45.8	0.76
*Comorbidities*			
COVID-19 actual	14.2	20.6	0.50
Ischaemic heart disease	12.7	4.2	0.17
Heart failure	17.9	16.7	1.00
Peripheral artery disease	15.8	16.7	0.83
Cerebrovascular disease	14.9	6.3	0.14
Dementia	12.8	31.3	**0**.**007**
COPD	16.4	12.5	0.52
Severe liver disease	11.9	12.5	1.00
Diabetes, with complications	6.0	16.7	**0**.**04**
Hemiplegia or tetraplegia	19.4	16.7	0.83
Chronic kidney failure	27.6	39.6	0.15
Dialysis	4.5	12.5	**0**.**06**
Obesity	11.2	12.5	0.80
Cancer	19.5	25.0	0.42
Haematological malignancy	7.5	8.3	0.85
AIDS	1.5	0	1.00
Transplant	6.0	6.3	1.00
Parenteral nutrition	12.4	22.7	0.10
Chemotherapy or radiotherapy	5.3	8.7	0.42
Acute respiratory failure	30.6	53.2	**0**.**004**
CCI	5.3 (3.2)	5.9 (2.2)	0.14
FI-Lab	0.33 (0.16)	0.66 (0.10)	**<0**.**0001**
CRP (mg/L)	118.2 (82.1)	149.2 (79.8)	**0**.**03**
PCT (ng/mL)	18.8 (38.0)	14.1 (22.8)	0.40

Numbers are reported as means (with standard deviation) for continuous variables and as percentages for categorical parameters, bold *P*-values denote statistically significant results (*P* < 0.05).

**Figure 1.**
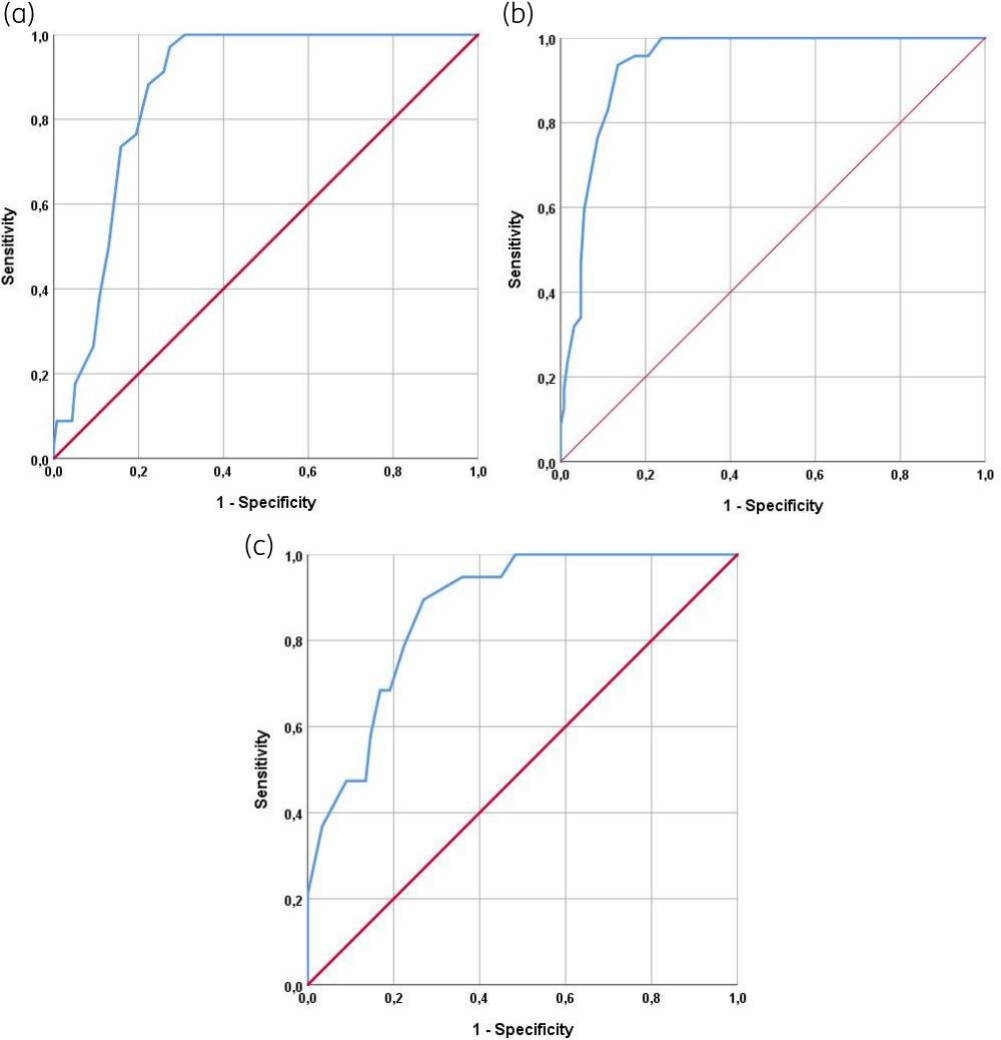
ROC curves of the FI-Lab in predicting mortality (a), 28 day mortality (b) and relapse (c).

The ability of FI-Lab to predict adverse outcomes was evaluated using ROC curve analysis (Table 3, Figure 1).

**Table 3.** Accuracy of FI-Lab in predicting negative outcomes in patients affected by KPC

Outcome	AUC	95%CI	*P* value	Sensitivity	Specificity
Mortality	0.94	0.91–0.97	<0.0001	100	76.2
28 day mortality	0.87	0.82–0.92	<0.0001	100	69.1
Relapse	0.87	0.80–0.94	<0.0001	79.2	77.3

Data are reported as areas under the curve with their 95% confidence intervals. The values for sensitivity and specificity were reported for the best cut-off points, using the Youden’s index.

In our cohort of patients with *K. pneumoniae* carbapenemase (KPC)-producing BSIs, the overall incidence of in-hospital mortality was 48% (48 out of 100 patients), while 28 day mortality occurred in 28% of cases. Additionally, relapse of infection was observed in 18% of patients. As shown in Table 3, the FI-Lab demonstrated excellent predictive ability for in-hospital mortality, with an AUC of 0.94 (95% CI: 0.91–0.97, *P* < 0.0001), 100% sensitivity and 76.2% specificity. For 28 day mortality, the AUC was 0.87 (95% CI: 0.82–0.92), also with 100% sensitivity and 69.1% specificity. Similarly, the FI-Lab showed good performance in predicting relapse, with an AUC of 0.87 (95% CI: 0.80–0.94), sensitivity of 79.2% and specificity of 77.3%.

In Table 4, the strength of association between FI-Lab and adverse outcomes was further confirmed through Cox regression analysis. A higher FI-Lab score was significantly associated with increased hazard of in-hospital mortality (HR: 2.07; 95% CI: 1.67–2.57; *P* < 0.0001), 28 day mortality (HR: 1.86; 95% CI: 1.47–2.35; *P* < 0.0001) and infection relapse (HR: 1.52; 95% CI: 1.05–2.21; *P* = 0.03). These findings suggest that FI-Lab is a strong and independent predictor of both short-term mortality and recurrence of CR-Kp BSI, offering a practical and objective tool for early risk stratification (Table 5).

**Table 4.** Association between FI-Lab in predicting negative outcomes in patients affected by KPC

Outcome	Number of events	HR	95% CI	*P* value
Mortality	48	2.07	1.67–2.57	<0.0001
28 day mortality	28	1.86	1.47–2.35	<0.0001
Relapse	18	1.52	1.05–2.21	0.03

Data are reported as hazard ratios (HRs) with their 95% confidence intervals (CIs) and correspondent *P* values, adjusted for age, sex, SOFA score and CCI.

**Table 5.** Association between antibiotic therapy in predicting negative outcomes in patients affected by KPC

Outcome	All sample			FI >0.46		
	HR	95% CI	*P* value	HR	95% CI	*P* value
Mortality	0.82	0.39–1.71	0.59	0.52	0.25–0.95	**0**.**03**
28 day mortality	0.65	0.28–1.53	0.32	0.43	0.18–0.99	**0**.**04**
Relapse	0.26	0.06–0.97	**0**.**04**	0.11	0.01–0.85	**0**.**04**

Data are reported as hazard ratios (HRs) with their 95% confidence intervals (CIs) and correspondent *P* values, adjusted for age, sex, SOFA score and CCI. Bold *P*-values denote statistically significant results (*P* < 0.05).

Multivariate analyses confirmed that FI-Lab was an independent predictor of adverse outcomes (Table 3). For each 0.10-point increase in FI-Lab, the hazard ratio (HR) for mortality increased by (95% CI: 1.67–2.57, *P* < 0.0001).

A similar association was observed for 28 day mortality (HR = 1.86, 95% CI: 1.47–2.35, *P* < 0.0001) and relapse (HR = 1.52, 95% CI: 1.05–2.21, *P* = 0.03). These findings were adjusted for age, sex, comorbidities (measured using the CCI) and SOFA score.

## Discussion

BSIs caused by carbapenemase-resistant *K. pneumoniae* (CR-Kp) remain a significant clinical challenge due to their association with high morbidity and mortality rates, particularly among older frail patients.^2,4,23^ Despite advancements in therapeutic options, including the use of novel beta-lactam beta-lactamase inhibitors (BLBLIs) either in monotherapy or in combination with other antibiotic classes for synergistic effects, treatment failures remain a pressing concern.^5,6,29,30^ Understanding the risk factors associated with poor outcomes is essential to improve patient management. In our cohort of 182 patients with CR-Kp BSI, the all-cause mortality rate was 26.3%.

The FI-Lab was a strong determinant of survival. The FI-Lab, derived from routine laboratory data available within the first 96 h of admission, serves as a rapid and practical tool for frailty assessment. In fact, this score, derived from simple laboratory examinations and in settings with an informatic system, needs only a few seconds to be created.^19^ Its simplicity and applicability across diverse clinical settings makes it a valuable first-line screening tool for physiological vulnerability.^19^ The predictive performance of the FI-Lab in our study was excellent, as evidenced by its high discriminatory power for mortality (AUC = 0.94), 28 day mortality (AUC = 0.87) and relapse (AUC = 0.87). Multivariate analyses confirmed that FI-Lab independently predicted adverse outcomes, even after adjusting for age, sex, comorbidities (CCI) and SOFA score. Unlike traditional comorbidity indices or organ dysfunction scores, FI-Lab offers a snapshot of systemic physiological reserve based solely on routine laboratory values, making it a practical and scalable tool for bedside risk stratification. Its strong predictive performance across multiple clinical endpoints suggests that FI-Lab could serve as a valuable addition to current prognostic models for patients with multidrug-resistant infections, particularly when functional or clinical frailty assessments are not feasible.

Despite its strengths, our study has several limitations. The absence of stratification between monotherapy and combination therapy limits the granularity of our findings, particularly given the growing use of BLBLIs in combination with other antibiotic classes.^3,6,7,11,22,31,32^ Additionally, the lack of detailed clinical data on the CR-Kp BSI episodes, including the specific site of infection (e.g. pneumonia, intra-abdominal or catheter-related), the ward of acquisition (ICU versus general ward) and polymicrobial infections—critical factors affecting antibiotic penetration and treatment success can significantly influence patient outcomes and may affect the applicability and interpretation of FI-Lab in this context.^13,33–35^ Delayed initiation of appropriate therapy, a known determinant of poor outcomes, also warrants further investigation.^36^ Future studies should aim to incorporate these variables and evaluate the FI-Lab in larger, more diverse cohorts.

### Conclusion

Frailty, as measured by the FI-Lab, is a powerful and independent predictor of poor outcomes in patients with BSIs caused by carbapenemase-resistant *K. pneumoniae*. Higher FI-Lab scores are strongly associated with increased mortality and relapse rates. The FI-Lab, with its simplicity and accessibility, represents an invaluable tool for early risk stratification, particularly in frail patients (FI-Lab >0.46), who demonstrate a markedly higher risk of adverse outcomes.

Further studies involving larger and more heterogeneous populations are essential to validate the FI-Lab and explore its potential as a predictive tool across different clinical scenarios. These studies should also address the influence of therapy timing, infection site and treatment combinations to optimize patient outcomes.

## Data Availability

All data generated or analysed during this study are included in this published article (and its supplementary information files).
